# Complete Genome Sequence of a Novel Bacterium within the Family *Rhodocyclaceae* That Degrades Polycyclic Aromatic Hydrocarbons

**DOI:** 10.1128/genomeA.00251-15

**Published:** 2015-04-09

**Authors:** David R. Singleton, Allison N. Dickey, Elizabeth H. Scholl, Fred A. Wright, Michael D. Aitken

**Affiliations:** a Department of Environmental Sciences and Engineering, University of North Carolina, Gillings School of Global Public Health, Chapel Hill, North Carolina, USA; b Bioinformatics Research Center, North Carolina State University, Raleigh, North Carolina, USA

## Abstract

A polycyclic aromatic hydrocarbon-degrading bacterium designated strain Ca6, a member of the family *Rhodocyclaceae* and a representative of the uncharacterized pyrene group 1 (PG1), was isolated and its genome sequenced. The presence of several genes suspected to be associated with PG1 was confirmed, and additional genes for aromatic compound metabolism were detected.

## GENOME ANNOUNCEMENT

Polycyclic aromatic hydrocarbons (PAHs) are ubiquitous chemicals that, when concentrated at contaminated sites, can pose a serious threat to human and environmental health. Of particular concern are high-molecular-weight PAHs of four or more aromatic rings that are potentially both more carcinogenic and resistant to microbial degradation than are their low-molecular-weight counterparts (1, 2). A prior stable-isotope probing study with the four-ring PAH pyrene revealed DNA sequences from several groups of uncharacterized proteobacteria (3). One predominant group of sequences within the betaproteobacterial family *Rhodocyclaceae*, designated pyrene group 1 (PG1), was later also implicated in the degradation of phenanthrene (4). A previously constructed metagenome from a mixed culture dominated by PG1 contained eight different ring-hydroxylating dioxygenase (RHD) genes predicted to code for enzymes involved in the initial step of aerobic PAH metabolism (5). Six of those RHDs heterologously expressed in *Escherichia coli* demonstrated PAH transformation capabilities (5). We subsequently isolated a member of PG1, designated strain Ca6, and its genome was sequenced.

DNA was extracted from a culture of Ca6 grown with pyrene using a modification of the FastDNA spin kit for soil (MP Biomedicals, Santa Ana, CA). The PacBio RSII platform, utilizing 3 single-molecule real-time (SMRT) cells, was used to acquire the raw sequence reads at the University of North Carolina High-Throughput Sequencing Facility. The reads were *de novo* assembled into a single contig with SMRT Analysis 2.2 using HGAP3, and the contig was polished using Quiver (6). Significant overlap between the ends indicated that the molecule was circular, and the Minimus2 assembler was used to circularize a split contig (7). Final polishing was performed with Quiver, and the resulting consensus sequence had an accuracy of >99.999%.

The finalized circular contig comprised 2,934,611 bp, with an average coverage of 228×. This chromosome was annotated by both the Department of Energy Joint Genomics Institute (JGI) Integrated Microbial Genomes system and the NCBI Prokaryotic Genome Annotation Pipeline. The JGI annotation predicted 2,902 genes, with 2,847 protein-coding genes, two copies of the rRNA operons, and 45 tRNA genes. The G+C content of the chromosome is 55.14%. The closest described relative is *Sulfuritalea hydrogenivorans* strain sk43H (94% 16S rRNA gene identity).

The predicted proteins were submitted to AromaDeg, a database for the identification of genes associated with the degradation of aromatic compounds (8). All eight RHD genes previously detected in a metagenome were present in the genome of Ca6. AromaDeg additionally identified four genes potentially associated with benzene or benzoate degradation, three and six putative extradiol dioxygenase genes associated with monocyclic and bicyclic compounds, respectively, two extradiol dioxygenases in the protocatechuate family, and a number of additional Rieske-type proteins.

Strain Ca6 is currently being characterized as belonging to a novel genus and species. Gene sequences highly similar to strain Ca6 have been detected in pristine and contaminated sites worldwide, so this organism represents a possible prominent degrader of hazardous aromatic compounds in both natural and engineered systems.

### Nucleotide sequence accession number.

The NCBI-annotated genome of Ca6 was deposited in GenBank under the accession no. CP010554. The JGI-annotated version is available at the DOE JGI-IMG website under the name “*Rhodocyclaceae* bacterium PG1-Ca6.”
